# Loneliness and Health Indicators in Middle-Aged and Older Females and Males

**DOI:** 10.3389/fnbeh.2022.809733

**Published:** 2022-03-10

**Authors:** Isabel Crespo-Sanmiguel, Mariola Zapater-Fajarí, Ruth Garrido-Chaves, Vanesa Hidalgo, Alicia Salvador

**Affiliations:** ^1^Laboratory of Social Cognitive Neuroscience, Psychobiology-IDOCAL, University of Valencia, Valencia, Spain; ^2^Department of Psychology and Sociology, Area of Psychobiology, IIS Aragón, University of Zaragoza, Teruel, Spain

**Keywords:** loneliness, subjective health, stress, HPA axis functioning, cortisol, aging

## Abstract

Loneliness is a complex and uncomfortable feeling that results from the perception of a lack of desired personal and social ties. Loneliness is accentuated with aging. It has been related to a wide range of objective and subjective health indicators and is a risk factor for morbidity and mortality. One of the proposed underlying mechanisms through which loneliness affects health is the dysregulation of the Hypothalamic-Pituitary-Adrenal (HPA) axis. However, the relationship between loneliness and cortisol, the main product of the HPA axis, is unclear and requires more research. The aims of this cross-sectional study were to investigate the relationships between loneliness, subjective health, and cortisol indexes, taking the sex into account, and investigate whether the HPA axis mediates the relationship between loneliness and subjective health. For this purpose, 79 participants (between 55 and 75 years old) completed several scales on loneliness, depression, perceived stress, psychological and physical health, and social relationships. Various salivary cortisol measurements were obtained on two consecutive days. The initial results showed that loneliness was related to psychological and physical health in the mixed-sex sample. However, when covariates were introduced, loneliness was only associated with psychological health in males. In addition, the cortisol indexes employed were not related to loneliness and did not mediate the relationship between loneliness and subjective health. Hence, we did not find a relevant role of the HPA axis in the association between loneliness and subjective health. More severe perceptions of loneliness would probably be necessary to detect this role. Overall, these results also show that the expected negative outcomes of loneliness associated with aging can be countered by an active life that can compensate for the natural losses experienced with age or at least delay these negative outcomes. Finally, some sex differences were found, in line with other studies, which warrants further examination of social variables and dimensions related to gender in future research.

## Introduction

Humans are essentially social beings. From birth, we need an attachment figure in order to develop, survive, and understand how the social universe and personal ties work (Bowlby, 1973, 1980; Fonagy and Luyten, 2018). During the rest of life, we have the need to be integrated into a social network (Baumeister and Leary, 1995). When this need is not fulfilled, loneliness appears, the painful feeling that accompanies the perception of a lack of desired personal and social relationships (Peplau and Perlman, 1982). Hence, loneliness can be understood as a potent psychosocial stressor (Cacioppo et al., 2003; Hawkley and Cacioppo, 2003). In addition, loneliness increases with age and is usually associated with adverse health outcomes (Luo et al., 2012; Victor and Yang, 2012; Holt-Lunstad et al., 2015; Cohen-Mansfield et al., 2016; Richard et al., 2017).

Thus, loneliness has been related to self-reported psychological and physical health issues, such as poor health and low life satisfaction (Losada et al., 2012; Tomstad et al., 2017), subjective memory complaints (Montejo et al., 2019), lower self-esteem (Wagner et al., 2015), depression (Aylaz et al., 2012; Ge et al., 2017), mobility characteristics (Van Den Berg et al., 2016), sleep disorders (Shankar, 2020), or more doctor visits (Beutel et al., 2017; Richard et al., 2017). In addition, loneliness plays a mediating role between early life stress and perceived stress in adulthood (Crespo-Sanmiguel et al., 2021). These health issues can be explained by the fact that loneliness acts as a stressor, activating the stress response *via* hypothalamic-pituitary-adrenal (HPA) axis functioning through the action of cortisol (Hawkley and Cacioppo, 2003; Steptoe et al., 2004; O’Connor et al., 2021). In this regard, a dysregulation of this axis has been associated with different harmful health issues (Fries et al., 2009; Adam et al., 2017).

Several studies have investigated the relationship between loneliness, the cortisol awakening response (CAR), and the diurnal cortisol slope (DCS) in aging, but with inconsistent results. In middle-aged adults, loneliness was found to be associated with CAR (Steptoe et al., 2004; Adam et al., 2006; Okamura et al., 2011), but no differences were found in CAR or DCS based on loneliness in older males and females (Schutter et al., 2017, 2020). However, in older people, a flatter DCS was found in lonely people in comparison with non-lonely individuals (Cole et al., 2007). In a previous study, we found that loneliness was positively associated with bedtime cortisol levels, but not with awakening cortisol or the DCS (Montoliu et al., 2019). As far as we know, our previous study was the first one to study the possible association between loneliness and bedtime cortisol levels. However, we could not test subjective health in relation to loneliness. Thus, the association between loneliness, cortisol indexes, and subjective health requires further research.

Sex differences have been found in some research on loneliness and its effects on subjective health and HPA axis functioning. Specifically, Zebhauser et al. (2014) reported that loneliness had a higher impact on subjective psychological health in males than in females. Furthermore, a dysregulation of the HPA axis, with a diminished CAR and flatter DCS, has been found in lonely married males, but not in their female counterparts (Johar et al., 2021). However, other studies did not find sex differences in the relationship between loneliness and subjective health (Richard et al., 2017), the DCS, or bedtime cortisol (Montoliu et al., 2019).

Given that loneliness can be understood as a stressor, we first aimed to study whether loneliness is associated with subjective psychological and physical health and HPA axis functioning in late middle-aged and early older people (older people henceforth). Second, we also aimed to explore whether these results differ depending on sex. Finally, we investigated whether HPA axis functioning is a mechanism underlying the relationship between loneliness and subjective health. We hypothesized that there would be a negative association between loneliness and subjective psychological and physical health (Beutel et al., 2017; Richard et al., 2017), and we expected to confirm the association between loneliness and cortisol indexes found previously (Montoliu et al., 2019). Finally, despite the heterogeneity in the findings, we expected to find clearer relationships in males than in females.

## Materials and Methods

### Participants

Participants were recruited from a study program for people over 55 years old at the University of Valencia (Spain). This program, called “La Nau Gran,” is a 3-year education program with basic modules from one of the different official degrees. The students are not issued an official degree, and they do not share the subjects with the regulated degree students. However, this program allows middle-aged and older people who want to learn and continue to grow to access the university as students. Two researchers from the laboratory came to these lessons to offer students the chance to participate in the research. Information on the type of data collected, the duration of the session, and the location of the laboratory was provided. Interested students provided their contact information. Participants who were not excluded, based on the exclusion criteria in a telephone interview, were given an appointment to attend an individual session at the Laboratory of Social Cognitive Neuroscience, of the University of Valencia.

Exclusion criteria were: being outside the age range from 55 to 75 years old; having diseases and disorders that interfere with daily wellbeing, such as endocrine (e.g., Type II diabetes), neurological (e.g., *epilepsy*), psychiatric (e.g., *personality and psychotic disorders or depression)*, or other diseases (*cancer, cerebrovascular diseases or chronic pain)*; using a medication such as glucocorticoids, anxiolytics, antidepressants, or other medications that can interfere directly with emotional, endocrinological, or cognitive functioning; having been under general anesthesia in the past 12 months; smoking more than 10 cigarettes a day, alcohol or other drug abuse; and the presence of a stressful life event during the past year, such as the death of the spouse, the appearance of a major disease, or any other event that had affected them in a significant way.

### Procedure

The current study follows an observational and cross-sectional design. Experimental sessions, which lasted approximately 1 h, had three different schedules (at 10 a.m, 12 p.m, and 4 p.m). Both the schedule and the sex of the participants were counterbalanced, so that the number of participants and the number of males and females who attended each session were similar. During the session, a general questionnaire about sociodemographic information and questionnaires about loneliness, subjective health, social relationships, depressive symptomatology, and stress perception were filled out. Furthermore, weight and height measurements were taken to calculate the body mass index (BMI). The experimenter explained verbally to the participants how and when they had to collect salivary cortisol samples at home. In addition, written instructions were given to participants, attached to a diary where they could provide the collection times of the salivary samples. Within 3 days after the session, participants returned to the laboratory to bring the samples. The protocol was approved by the Research Ethics Committee of the University of Valencia (Code: 1034878) and written according to the Declaration of Helsinki. All participants read and signed the informed consent. The period of the data collection was between January 2018 and January 2020.

### Questionnaires

#### Socioeconomic Status

We assessed the socioeconomic status with the nine-rung social ladder (Adler and Stewart, 2007). A ladder with nine rungs is presented by explaining that the highest rungs would contain the people in our country with the highest standing, that is, with a lot of money, a good education, and the best jobs. In contrast, the lowest rungs would contain the poorest people with less education and worse jobs or no job. Participants are asked the following question: Where would you place yourself on this ladder?

#### Loneliness

We used the Spanish adaptation (Vázquez Morejón and Jiménez García-Bóveda, 1994) of the revised UCLA loneliness scale (R-UCLA) (Russell et al., 1980) to assess loneliness. This scale contains 20 items rated on a 4-point Likert scale ranging from 1 (never) to 4 (often), obtaining a total score ranging from 20 (low) to 80 (high). The Cronbach’s α in our study was 0.887.

#### Subjective Psychological and Physical Health and Social Relationships

We used the three domains of the Spanish version of the World Health Organization Quality of Life Short Form Survey (Carrasco, 1998), created by the WHOQOL Group (1998), to assess the perception of: (i) psychological health (e.g., self-esteem, positive and negative feelings, or capacity for concentration); (ii) physical health (e.g., mobility, activities of daily living, sleep and rest, or pain and discomfort); and (iii) social relationships (e.g., social support or sexual activity). The subscales have 6, 7, and 3 items, respectively, with a 5-point Likert response scale ranging from 1 to 5. The scores were transformed to a scale from 0 to 100, where higher scores represent perceptions of better health. The internal consistency (Cronbach’s α) for the psychological health scale was 0.763, for the physical health scale 0.623, and for the social relationships scale 0.708.

#### Depressive Symptomatology

We used the Spanish version (Sanz et al., 2003) of the Beck Depression Inventory-II (BDI-II; Beck et al., 1996) to assess depressive symptomatology. This test consists of 21 items, with a response scale ranging from 0 to 3, that evaluate the symptoms of depression (emotional, cognitive, somatic, and motivational) in the past month. Scores range from 0 to 63, where higher scores are interpreted as higher symptomatology. The internal consistency (Cronbach’s α) of this scale was 0.868.

#### Perceived Stress

The degree to which people perceived their lives as stressful and overloaded was evaluated using the Spanish version (Remor, 2006) of the 14-item Perceived Stress Scale (PSS-14; Cohen et al., 1983). Participants had to answer using a 5-point Likert scale ranging from 0 (never) to 4 (very often), and total scores range from 0 to 56, with higher scores indicating higher stress perception. The evaluation refers to the past month. The internal consistency (Cronbach’s α) of this scale in this study was 0.801.

### Cortisol Measurements

Ten salivary samples were collected to assess participants’ diurnal cortisol levels using Salivettes (Sarstedt, Rommelsdorf, Germany). The saliva samples were collected by participants immediately, + 15, + 30 and + 45 min after awakening and before sleep on two consecutive days in their natural environment and without disturbing their usual daily activities. Participants were instructed to keep the cotton in their mouth for exactly 2 min and then store it in the refrigerator until they took it to the laboratory. They returned the samples as soon as possible, with 3 days after their collection being the maximum time. Once in the laboratory, the saliva samples were centrifuged for 15 min at 4,000 rpm, resulting in a clear supernatant of low viscosity that was stored at −80^°^C until analyses were performed. The ELISA kit from Salimetrics (Newmarket, United Kingdom) was used to determine the cortisol concentrations.

For each participant, all samples were measured in duplicate and analyzed in the same trial. The assay sensitivity and the inter- and intra- assay variation coefficients of raw densities were below 10%. Salivary cortisol levels were determined in the Laboratory of Social Cognitive Neuroscience (Valencia, Spain).

### Statistical Analyses

Because the cortisol levels did not follow a normal distribution, they were log transformed. We used three cortisol indexes: (i) the CAR, a dynamic measure of post-awakening cortisol secretion, calculated from cortisol samples taken 0, + 15, + 30, and + 45 min after awakening following the trapezoidal formula for the area under the curve with respect to the increase (AUCi; see Pruessner et al., 2003); (ii) the DCS, which is calculated as awakening cortisol minus bedtime cortisol and reflects the diurnal decline in cortisol levels; and (iii) bedtime cortisol, which reflects cortisol levels immediately before going to sleep. To calculate each cortisol index, the mean for both days was used, and for participants who had missing cortisol data from one of the 2 days (CAR: *n* = 24, DCS: *n* = 7, bedtime: *n* = 6), we used the data from the day they were available. To study the effect of CAR compliance, we reran the analysis, excluding 12 participants (15.19%) who were outside the recommended strict time window, that is, with a 5-min delay (Stalder et al., 2016). These results are reported as Supplementary Materials.

To evaluate sex differences, Student’s *t*-tests for independent samples were performed for age, socioeconomic status, BMI, loneliness, psychological and physical health, cortisol indexes, social relationships, depressive symptomatology, perceived stress, and hours spent with their children. In addition, X^2^ were performed for educational level, marital status, and number of children, and Z analysis was performed for the statistically significant results of X^2^. To investigate whether loneliness was related to subjective psychological and physical health and HPA axis functioning, linear regression analyses were performed. In these analyses, loneliness was the independent variable, and subjective psychological and physical health and the cortisol indexes were the dependent variables. Additionally, we tested whether these relationships varied depending on sex by performing moderation analyses. Thus, loneliness was the independent variable, sex was the moderator variable, and subjective health (psychological and physical) and endocrine indicators (cortisol indexes) were the dependent variables. Both linear regression and moderation analyses were performed separately for each dependent variable. Finally, we tested whether the HPA axis mediates the relationship between loneliness and subjective psychological and physical health by performing mediation analyses. Thus, loneliness was the independent variable, the cortisol indexes were the mediators, and the two types of subjective health (psychological and physical) were the dependent variables.

Because loneliness has been widely related to depressive symptomatology (Erzen and Çikrikci, 2018) and most of the studies on loneliness and cortisol control depression in the analyses (Schutter et al., 2017; Montoliu et al., 2019; Johar et al., 2021), we included depressive symptomatology as covariate in the regression, moderation, and mediation analyses. Moreover, for the regression, moderation, and mediation analyses that include the CAR, we used time of awakening and cortisol levels immediately after awakening as covariates, as in Stalder et al. (2016). Furthermore, Pearson’s correlations were performed between the sociodemographic variables and loneliness, subjective psychological and physical health, and the cortisol indexes. The sociodemographic variables that were significantly related to these factors were used as covariates in the main analyses (regression, moderation, and mediation analyses). Thus, in all the main analyses, in addition to depressive symptomatology, age was controlled because it was positively related to loneliness in our sample. Likewise, socioeconomic status was a covariate in the main analyses that included subjective physical health, due to their relationship. BMI was a covariate in the main analyses that included bedtime cortisol, due to their relationship. In addition, due to the sex differences in marital status and in BMI, these variables were included as covariates in all the moderation analyses.

Multivariate outliers were considered those that deviated from the mean (± 3 *SD*), and standardized residuals were used to detect them. Specifically, there was an outlier in the main analyses that included the CAR. No collinearity issues were detected for the variables included in the main analyses, indicated by tolerance values > 0.1. One piece of data was missing for BMI and for level of studies, three for CAR and DCS, two for bedtime cortisol, thirteen for the time of awakening, and five for the hours per week spent with their children. Consequently, the number of participants in the different analyses varies.

We estimated a sample size of *N* = 55 to obtain a medium effect size (*f*^2^ = 0.15, α = 0.05 and power = 0.80), calculated using the G*Power (Faul et al., 2007). Thus, our sample size (*N* = 79) is adequate because in the recruitment we anticipated possible missing data. The bootstrap technique used for moderation and mediation analysis uses the original sample size as a miniature representation that is randomly replaced and resampled, which increases the statistical power and solves the problem of having a relatively small sample (Hayes, 2017).

To carry out all the statistical analyses, version 25.0 of SPSS was used. All *p*-values were two-tailed, and the level of significance was taken as *p* < 0.05. To test moderated and mediated regression effects, we used PROCESS 3.4 for SPSS (Model 1) and Z scores. PROCESS uses bootstrapped bias-corrected 95% confidence intervals with 5,000 bootstrapped samples in order to determine the significance of the interaction effect in the moderation analysis and the significance of the indirect effect in the mediation analysis. When the confidence interval for the interaction effect (moderation analysis) or the indirect effect (mediation analysis) did not include zero, it was interpreted that there was a significant interaction/indirect effect (Hayes, 2017).

## Results

### Descriptive Analyses

The sessions contained 82 participants, but three participants were eliminated due to missing data on the loneliness, depression, or health questionnaires. The final sample was composed of 79 participants (39 males, 40 females) with ages ranging from 55 to 75 years old (*M* = 64.481, *SD* = 5.563). Participants reported a medium-high socioeconomic status, medium-high levels of satisfaction with their social relationships, and low levels of depressive symptomatology and perceived stress. More than half the participants (57.7%) had university studies, and 52 (65.8%) were married. All the females were post-menopausal. There were no sex differences in loneliness [*t*(77) = 1.273, *p* = 0.207], subjective psychological [*t*(77) = 1.042, *p* = 0.301] or physical health [*t*(77) = −0.898, *p* = 0.372], or the cortisol indexes [CAR: *t*(64) = −0.041, *p* = 0.968; DCS: *t*(74) = 0.094, *p* = 926; bedtime: *t*(75) = 0.600, *p* = 0.551]. Significant differences were only found in BMI [*t*(76) = 3.999 *p* < 0.001] and marital status [*X*^2^(4) = 9.691, *p* = 0.046], with a higher proportion of married males compared to married females and of widowed females compared to widowed males. Sample characteristics are described in Table 1.

**TABLE 1:** Characteristics of the total sample and for males and females.

	Total (*N* = 79)	Males (*N* = 39)	Females (*N* = 40)	*t* (*p*)/*X*^2^(*p*)
Age	64.481 (5.563)	65.385 (5.683)	63.600 (5.368)	1.435 (0.155)
**Educational level:**				6.047 (0.302)
Primary school or less	10.3	7.9	12.5	
Secondary school	32.1	23.7	40.0	
Graduate	56.4	65.7	47.5	
Ph.D.	1.3	2.6	0	
**Marital status:**				9.691 (0.046)
Single	8.9	2.6	15.0	
Married	65.8	76.9	55.0	
Divorced	15.2	18.0	12.5	
Widower	10.1	2.6	17.5	
SES	6.114 (1.577)	6.205 (1.592)	6.025 (1.577)	0.505 (0.615)
BMI	26.658 (4.039)	28.331 (3.594)	24.985 (3.794)	3.999 (< 0.001)
Loneliness	35.810 (7.172)	36.846 (7.580)	34.800 (6.692)	1.273 (0.207)
Psychological health	65.722 (10.774)	67.000 (11.381)	64.475 (10.135)	1.042 (0.301)
Physical health	69.886 (11.706)	68.692 (9.134)	71.050 (13.782)	−0.894 (0.372)
**Cortisol indexes**				
CAR	0.296 (0.568)	0.293 (0.567)	0.299 (0.579)	−0.041 (0.968)
DCS	0.673 (0.326)	0.677 (0.293)	0.670 (0.361)	0.094 (0.926)
Bedtime levels	0.109 (0.242)	0.125 (0.201)	0.092 (0.280)	0.600 (0.551)
Depressive symptomatology	6.025 (6.006)	5.051 (4.530)	6.975 (7.091)	−1.433 (0.156)
Perceived stress	17.620 (6.779)	16.667 (7.317)	18.550 (6.160)	−1.239(0.219)
Social relationships	62.380 (16.765)	62.769 (15.727)	62.000 (17.911)	203 (0.840)
**Number of sons:**				2.508 (0.643)
0	6.3	2.6	10.0	
1	12.7	12.8	12.5	
2	63.3	69.2	57.5	
3	12.7	10.3	15.0	
4	5.1	5.1	5.0	
Time with sons^a^	9.041 (11.111)	8.158 (10.566)	9.972 (11.736)	−0.700 (0.486)

*Note. SES, Subjective socioeconomic status; BMI, Body Mass Index. ^a^Measured in hours per week. Sex differences in educational level, marital status and number of sons are expressed as percentages and analyzed with Chi-square tests. The means and standard deviations of other variables are shown and were analyzed with Student’s t-test.*

### Pearson’s Correlation Analyses

In our sample, loneliness increased with age (*p* = 0.001) and was negatively related to subjective psychological health (*p* = 0.011) and physical health (*p* = 0.038), but it was not significantly associated with the CAR (*p* = 0.710), DCS (*p* = 0.433), and bedtime (*p* = 0.950) cortisol indexes. Moreover, loneliness was negatively related to satisfaction with their relationships (*p* < 0.001) and to the hours per week they spent with their children (*p* = 0.006). However, loneliness was not significantly related to depressive symptomatology (*p* = 0.096) (Table 2).

**TABLE 2:** Pearson’s correlations.

	2	3	4	5	6	7	8	9	10	11	12	13	14	15
1. Age	0.213	0.247*	–0.044	0.376**	–0.046	–0.119	0.110	–0.018	–0.106	0.100	0.027	−0.222*	0.190	−0.329**
2. Educational level		0.234*	0.048	0.168	0.031	0.048	–0.021	–0.087	0.130	−0.269*	–0.006	−0.257*	–0.138	0.058
3. SES			–0.040	0.056	0.083	0.238*	0.109	–0.051	0.042	–0.148	–0.112	–0.028	0.233*	–0.014
4. BMI				0.044	0.032	–0.129	–0.096	–0.161	0.235*	–0.108	0.065	0.023	0.027	0.012
5. Loneliness					−0.284*	−0.234*	0.047	–0.091	0.007	0.189	0.139	−0.484**	–0.039	−0.315**
6. Psychological health						0.354**	0.069	–0.069	–0.047	−0.464**	−0.262*	0.355**	0.046	0.337**
7. Physical health							0.054	–0.041	0.114	−0.374**	−0.348**	0.325**	0.058	0.000
8. CAR								−0.424**	–0.038	0.039	–0.143	–0.129	0.003	0.099
9. DCS									−0.679**	0.179	0.005	0.114	0.012	0.010
10. Bedtime										–0.159	0.039	–0.040	0.019	0.015
11. Depressive sympt.											0.453**	−0.245*	–0.045	–0.101
12. Perceived stress												–0.220	−0.276*	0.013
13. Social relationships													0.814	–0.006
14. Number of sons														–0.056
15. Time sons^a^														

Note. SES, Subjective socioeconomic status; BMI, Body Mass Index; CAR, Cortisol awakening response; DCS, diurnal cortisol slope; Depressive sympt, Depressive symptomatology. ^a^Measured in hours per week. *p < 0.05 and **p < 0.01.

### Adjusted Regression Analyses

Results of adjusted regression analyses confirmed the significant negative relationship between loneliness and subjective psychological health (*p* = 0.034). However, when covariates were included, the relationship between loneliness and subjective physical health became non-significant (*p* = 0.167).^1^ The relationship between loneliness and the cortisol indexes was not significant either (all *p* > 0.291) (Table 3).

**TABLE 3:** Adjusted regression analyses with loneliness as a predictor and the psychological and physical health and cortisol indexes as dependent variables.

	Loneliness				
	***R*^2^ adjusted**	***R*^2^ change**	**Beta**	***p***	***N***

Psychological health	0.232	0.046	–0.235	0.034*	79
Physical health	0.171	0.021	–0.158	0.167	79
CAR	0.332	0.011	–0.110	0.319	65
DCS	0.009	0.015	–0.135	0.291	76
Bedtime cortisol	0.029	0.004	0.066	0.602	76

Note. CAR, cortisol awakening response; DCS, diurnal cortisol slope. Controlled by depressive symptomatology and age. In addition, socioeconomic status for physical health, time of awakening and cortisol levels immediately after awakening for CAR and body mass index for bedtime levels. *p < 0.05.

### Moderation Analyses

Results of the moderation analyses showed a significant interaction effect of sex in the relationship between loneliness and subjective psychological health (*p* < 0.001). Thus, loneliness was negatively related to subjective psychological health in males (*p* < 0.001), but not in females (*p* = 0.263). No interaction effect of sex was found in the relationships between loneliness and subjective physical health (*p* = 0.697). Additionally, sex did not moderate the relationship between loneliness and the cortisol indexes (all *p* > 0.274) (Table 4).

**TABLE 4:** Adjusted moderation analyses with loneliness as a predictor and psychological and physical health and cortisol indexes as dependent variables in males and females.

*Dependent variable: Psychological health*						
**ΔR2 interaction = 0.130 *F* = 15.361, df (1,2) = 1, 70 *p* < 0.001 LLCI = 0.183 ULCI = 0.563**						

**Sex**	**Effect**	**SE**	***t***	***p***	**LLCI**	**ULCI**

Males	–0.581	0.136	–4.284	< 0.001**	–0.851	–0.310
Females	0.162	0.143	1.129	0.263	–0.124	0.447

***Dependent variable: Physical health***						

**ΔR2 interaction = 0.002 *F* = 0.153, df (1,2) = 1, 69 *p* = 0.697 LLCI = −0.173 ULCI = 0.258**						

**Sex**	**Effect**	**SE**	***t***	***p***	**LLCI**	**ULCI**

Males	–0.177	0.154	–1.155	0.252	–0.484	0.129
Females	–0.093	0.162	–0.576	0.567	–0.417	0.230

***Dependent variable: CAR(1 outlier)***						

**ΔR2 interaction = 0.011 *F* = 1.038, df (1,2) = 1, 54 *p* = 0.313 LLCI = −0.638 ULCI = 0.208**						

**Sex**	**Effect**	**SE**	***t***	***p***	**LLCI**	**ULCI**

Males	0.008	0.153	0.051	0.960	–0.299	0.315
Females	–0.207	0.152	–1.360	0.180	–0.513	0.098

***Dependent variable: DCS***						

**ΔR2 interaction = 0.001 *F* = 0.039, df (1,2) = 1, 67 *p* = 0.844 LLCI = −0.255 ULCI = 0.209**						

**Sex**	**Effect**	**SE**	***t***	***p***	**LLCI**	**ULCI**

Males	–0.093	0.165	–0.559	0.578	–0.423	0.238
Females	–0.138	0.176	–0.783	0.436	–0.489	0.214

***Dependent variable: Bedtime***						

**ΔR2 interaction = 0.016 *F* = 1.219, df (1,2) = 1, 68 *p* = 0.274 LLCI = −0.362 ULCI = 0.104**						

**Sex**	**Effect**	**SE**	***t***	***p***	**LLCI**	**ULCI**

Males	0.185	0.166	1.116	0.269	–0.146	0.517
Females	–0.071	0.177	–0.399	0.691	–0.424	0.283

Note. CAR, cortisol awakening response; DCS, diurnal cortisol slope. **p < 0.01. Values for quantitative moderators are the mean and plus/minus one SD from mean.

### Mediation Analyses

Mediation analyses revealed that the cortisol indexes did not mediate the relationship between loneliness and subjective health. Regarding the analyses with subjective psychological health as dependent variable, the indirect effect (i.e., effect of loneliness on subjective psychological health *via* cortisol indexes) was not significant for any cortisol indexes: CAR (IC 95% [−0.025, 0.076]), DCS (IC 95% [−0.026, 0.058]), and bedtime (IC 95% [−0.047, 0.030]) (Table 5). In the analysis with subjective physical health as dependent variable, the indirect effect (i.e., effect of loneliness on subjective physical health *via* cortisol indexes) was not significant for any of the cortisol indexes: CAR (IC 95% [−0.082, 0.016]), DCS (IC 95% [−0.038, 0.038]), and bedtime (IC 95% [−0.023, 0.050]) (Table 6).

**TABLE 5:** Adjusted mediation models of the relationship between loneliness as predictor and psychological health as dependent variables *via* cortisol indexes (CAR, DCS, and bedtime).

*Mediating variable: CAR (1 outlier)*						
	**Effect**	**SE**	***t***	***p***	**LLCI**	**ULCI**

Loneliness to CAR	–0.109	0.109	–1.005	0.319	–0.327	0.108
CAR to psychological health	–0.106	0.136	–0.776	0.441	–0.378	0.167
Indirect effect	0.012	0.026	–	–	–0.025	0.076
Total effect	–0.258	0.113	–2.275	0.027*	–0.484	–0.031
Direct effect	–0.269	0.115	–2.349	0.022*	–0.498	–0.040

***Mediating variable: DCS***						

	**Effect**	**SE**	***t***	***p***	**LLCI**	**ULCI**

Loneliness to DCS	–0.133	0.125	–1.064	0.291	–0.381	0.116
DCS to psychological health	–0.013	0.106	–0.125	0.901	–0.225	0.198
Indirect effect	0.002	0.020	–	–	–0.026	0.058
Total effect	–0.245	0.112	–2.197	0.031*	–0.468	–0.023
Direct effect	–0.247	0.113	–2.180	0.033*	–0.473	–0.021

***Mediating variable: bedtime levels***						

	**Effect**	**SE**	***t***	***p***	**LLCI**	**ULCI**

Loneliness to bedtime	0.065	0.123	0.523	0.602	–0.181	0.311
Bedtime to psychological health	–0.106	0.108	–0.981	0.330	–0.322	0.110
Indirect effect	–0.007	0.018	–	–	–0.047	0.030
Total effect	–0.241	0.112	–2.146	0.035*	–0.466	–0.017
Direct effect	–0.234	0.113	–2.081	0.041*	–0.459	–0.010

Note. CAR, cortisol awakening response; DCS, diurnal cortisol slope. *p < 0.05.

**TABLE 6:** Adjusted mediation models of the relationship between loneliness as predictor and physical health as dependent variables *via* cortisol indexes (CAR, DCS, and bedtime).

*Mediating variable: CAR (1 outlier)*						
	**Effect**	**SE**	***t***	***p***	**LLCI**	**ULCI**

Loneliness to CAR	–0.098	0.107	–0.915	0.364	–0.312	0.116
CAR to physical health	0.163	0.143	1.138	0.260	–0.123	0.448
Indirect effect	–0.016	0.025	–	–	–0.082	0.016
Total effect	–0.096	0.117	–0.826	0.412	–0.330	0.137
Direct effect	–0.080	0.117	–0.687	0.495	–0.315	0.154

***Mediating variable: DCS***						

	**Effect**	**SE**	***t***	***p***	**LLCI**	**ULCI**

Loneliness to DCS	–0.133	0.126	–1.058	0.294	–0.383	0.118
DCS to physical health	0.011	0.110	0.097	0.923	–0.208	0.229
Indirect effect	–0.001	0.018	–	–	–0.038	0.038
Total effect	–0.136	0.115	–1.182	0.241	–0.366	0.094
Direct effect	–0.135	0.117	–1.152	0.253	–0.368	0.099

***Mediating variable: bedtime levels***						
	**Effect**	**SE**	***t***	***p***	**LLCI**	**ULCI**

Loneliness to bedtime	0.065	0.124	0.523	0.602	–0.182	0.312
Bedtime to physical health	0.091	0.111	0.823	0.414	–0.130	0.313
Indirect effect	0.006	0.018	–	–	–0.023	0.050
Total effect	–0.126	0.115	–1.098	0.276	–0.355	0.103
Direct effect	–0.132	0.115	–1.145	0.256	–0.362	0.098

*Note. CAR, cortisol awakening response; DCS, diurnal cortisol slope.*

## Discussion

The aims of this study were, first, to test whether loneliness was related to subjective psychological and physical health indicators and HPA axis functioning (CAR, DCS, and bedtime cortisol) and, second, to analyze the role of sex in these relationships. Finally, we investigated whether HPA axis functioning was a mediator in the relationship between loneliness and subjective health. In the total sample, loneliness was correlated with psychological and physical health, but when these relationships were analyzed in more detail (including the pertinent covariates), loneliness did not appear to be associated with subjective physical health or the cortisol indexes, and the only relationship that remained was between loneliness and psychological health in males, but not in females.

Males with higher loneliness scores showed lower subjective psychological health. This finding is in line with a previous study showing that males tend to experience greater effects of loneliness on mental health (Zebhauser et al., 2014). These authors, based on Stevens (1995), proposed that the sex differences could be due to the fact that females have more settings where they can obtain social support, whereas males seek more social contact in the public spheres of organizations, where it is more difficult to find close personal contacts. However, this explanation is not supported by our results because there are no sex differences in satisfaction with social relationships. Although there are no sex differences in loneliness in previous literature (Maes et al., 2019) or in our sample, the association between loneliness and subjective psychological health could be explained by sex differences in experiences in interdependent relationships. It has been observed that females focus more on intimate and dyadic attachments (Baumeister and Sommer, 1997; Gardner and Gabriel, 2004) in which they can share their most personal and intimate experiences and, thus, strengthen their psychological wellbeing. In contrast, males focus more on the group (Hoza et al., 2000), where there is no space to share concerns. These differences are often related to masculinity (ideals and rules about what it means to be a man), manifested as difficulty in expressing their needs (Wide et al., 2011), a tendency to solve problems independently (Roy et al., 2017), and being less likely to request psychological support through services (Ogrodniczuk et al., 2016). These traits could influence the way loneliness affects older people differently depending on their sex. Therefore, males would benefit less from their relationships in terms of mental health. However, more research is needed to understand the underlying mechanisms that associate loneliness with psychological health in males but not in females.

Previous studies found that lonely people were more likely to report factors related to poor physical health, such as visits to medical doctors, more chronic diseases, or poor subjective health, among others (Richard et al., 2017). In our sample, loneliness was associated with subjective physical health, although the inclusion of depressive symptomatology as a covariate made the relationship non-significant. This could be explained by the fact that depressive symptomatology can aggravate the way people perceive their health (Wells et al., 1989; Gaynes et al., 2002). In line with this, depressive symptomatology is associated with more difficulty sleeping (Koffel and Watson, 2009) and a wide variety of somatic complaints, which also share biological pathways and neurotransmitters with depression (Bair et al., 2003). Our results are not consistent with Richard et al. (2017), who found an association between loneliness and self-reported physical health. However, Richard et al. (2017) included participants with non-specified chronic illnesses, and this worse state of health may have corresponded to lower subjective physical health (Idler and Benyamini, 1997; Benyamini and Idler, 1999; Benyamini et al., 2014; Elran-Barak et al., 2019). In the current study, due to the restrictive exclusion criteria followed, participants did not have any important diseases that seriously interfered with their wellbeing, and they did not take any medications that could indicate an initial phase of illness. These characteristics could explain the non-relationship found between loneliness and subjective physical health.

Regarding the lack of association between loneliness and the HPA axis indexes, this result agrees with a previous study that did not find a relationship between loneliness and CAR (Schutter et al., 2017), although Johar et al. (2021) found a diminished CAR in lonely married males. However, in this latter study, the relationship between CAR and loneliness in males was no longer significant when adjusting for sociodemographic covariates, depression, or awakening time. Therefore, our results are in line with those of Johar et al. (2021), suggesting that loneliness and CAR are not associated. Furthermore, although in the regressions separated by sex, the relationship was significant for males, the sex interaction of the relationship between loneliness and CAR was not significant.

We also failed to find a relationship between loneliness and DCS. This result agrees with other previous studies (Schutter et al., 2017, 2020; Montoliu et al., 2019), although it contrasts with studies that found a flattened DCS in married participants (Johar et al., 2021) and in a selected sample with extremely high loneliness scores (Cole et al., 2007). In both of these studies, the sample was composed of people who could suffer from alcohol abuse, smoking, or diabetes, which can influence the DCS (Adam et al., 2017). These health issues alone could contribute to the dysregulation of the HPA axis functioning and skew the association between loneliness and cortisol patterns. In addition, in Cole et al. (2007), although the saliva samples were collected reliably (on three consecutive days and at three different time points due to the longitudinal study design), the procedure used to classify the groups and the small number of participants in each group could explain the disparity in the results. Specifically, the groups were composed of 14 participants who consistently scored in the top 15% of the loneliness distribution throughout the study (high-lonely group; *N* = 6) and in the bottom 15% (low-lonely; *N* = 8). Thus, the analyses of group differences were performed with extreme scores and small samples.

Regarding the lack of relationship between loneliness and bedtime cortisol, the current results did not confirm our previous study (Montoliu et al., 2019), which found a positive association between them. Although it is true that participants in both studies showed similar loneliness scores, there were protective factors that we added in the current study and had not tested before. The characteristics of our participants represent optimal aging: no chronic diseases, a high socioeconomic level, low depressive symptomatology, low perceived stress, children they see frequently, and high satisfaction with social relationships. These circumstances could be acting as a buffer against stressors (Hawkley et al., 2008) such as loneliness and its endocrine effects, or they could even keep feelings of loneliness from appearing (Teater et al., 2021).

Finally, the results of the mediation analyses showed that the direct relationship between loneliness and psychological health was negative, confirming the regression analyses. However, the indirect effect that indicates whether HPA axis functioning is an underlying mechanism between loneliness and health was not found, contrary to what other authors suggested (Hawkley and Cacioppo, 2003; Steptoe et al., 2004). Considering all this, it is worth noting that loneliness is an experience with potentially adverse effects on psychological health, although more research is needed on what factors could influence the way loneliness affects subjective health in older people without severe perceptions of loneliness.

Some limitations should be considered when interpreting the results of this study. First, the cross-sectional design of the study makes it impossible to draw conclusions about causal relationships. Second, the internal consistency of the physical health scale shows low values, and so the results that include this scale should be confirmed in further studies. Moreover, to control possible confounders, this study had restrictive exclusion criteria, and only participants who were in good general health were included. Our participants have healthy characteristics and behaviors, and perhaps due to this, in our study loneliness was not related to the perception of physical health or to HPA axis indicators. Therefore, in future studies, these associations could be tested using longitudinal designs, other middle and older age ranges, and people with greater loneliness, for example, due to social circumstances or chronic diseases, such as older people with diabetes who report higher feelings of loneliness (Hackett et al., 2020).

Our findings support the view that loneliness is not associated with HPA dysregulation, but it is associated with subjective health, specifically psychological health in males. Subjective health is an important health measure because it predicts the evolution of health and life expectancy as well as or even better than objective health examinations (Miilunpalo et al., 1997; Helmer et al., 1999). Finally, males appeared to be more vulnerable to loneliness because loneliness was related to their subjective health, and so they could benefit from prevention procedures and follow-ups to avoid more severe psychological difficulties.

## Data Availability Statement

The raw data supporting the conclusions of this article will be made available by the authors, without undue reservation.

## Ethics Statement

The studies involving human participants were reviewed and approved by the Research Ethics Committee of the University of Valencia (Code: 1034878). The patients/participants provided their written informed consent to participate in this study.

## Author Contributions

IC-S participated in the data acquisition, managed the literature search, the statistical analyses, and with AS and VH interpreted the results, revised the literature, and wrote the manuscript. MZ-F and RG-C contributed to the data acquisition and management. All the authors contributed to and approved the final manuscript.

## Conflict of Interest

The authors declare that the research was conducted in the absence of any commercial or financial relationships that could be construed as a potential conflict of interest.

## Publisher’s Note

All claims expressed in this article are solely those of the authors and do not necessarily represent those of their affiliated organizations, or those of the publisher, the editors and the reviewers. Any product that may be evaluated in this article, or claim that may be made by its manufacturer, is not guaranteed or endorsed by the publisher.

## Footnotes

1 To study in more detail which covariate is influencing the change in the significance of the relationship between loneliness and physical health, we performed a stepwise regression including the covariates (age, socioeconomic status, and depressive symptomatology). Depressive symptomatology was the only covariate that remained in the model, and so it is the one that is modifying the statistical conclusion.
